# Supplementary material for: Optimizing delivery strategies for 3HP TB preventive treatment in Tanzania: A qualitative study on acceptability of family approach in HIV care and treatment centers
Source: PLOS Glob Public Health. 2024 Dec 17;4(12):e0003142. doi: 10.1371/journal.pgph.0003142 (PMC11651582; doi:10.1371/journal.pgph.0003142)
Supplement: Attachment — Submitted filename: Response to Reviewers.pdf [file pgph.0003142.s005.pdf]

THE UNITED REPUBLIC OF TANZANIA

MINISTRY OF HEALTH

NATIONAL INSTITUTE FOR MEDICAL RESEARCH

Date: 18th August 2024

Dear Editor(s)

The authors of this manuscript would like to thank you and the reviewers for dedicating time to review our work and for providing constructive feedback. Your input has significantly improved both our scientific writing and the content of the manuscript. Below is a thorough point-by-point response to each editorial point raised by reviewers. All revisions made to the manuscript are clearly indicated using tracked changes.

Reviewer #1: Dear co-authors, congratulations on a thorough manuscript addressing an important topic. Although I only have three comments (outlined below), I hope that you are able to use these to revise more generally for clarity and precision. The manuscript overall feels unnecessarily long, with many of the quotes not adding much value to the actual finding they represent.

> *Response: This is very valid. Thank you for pointing this out. We have deleted redundant quotes and modified the text to reduce the overall length of the manuscript.*

1. The general writing requires editing. It is beyond the scope of a reviewer and the journal to do this. But it is needed. Here are a few examples from just a short section. These occur throughout: line 418 - 'The PLHIV viewed using a closed one'; what does that mean? A person who is close to the PLHIV? Close is not the same as closed. And a 'close one' still reads as quite odd.

Headquarters: 3 Barack Obama Drive, P.O. Box 9653, 11101 Dar es Salaam, Tanzania,
Phone: +255222121400, Email: info@nimr.or.tz, Website: www.nimr.or.tz

NIMR Amani, P.O. Box 81, Muheza, Tanga, Tanzania, Email: amani@nimr.or.tz

NIMR Dodoma, Benjamin Mkapa Hospital, P.O. Box 805, 41119 Dodoma, Tanzania, Email: dodoma@nimr.or.tz

NIMR Mbeya, Hospital Hill Rd, P.O. Box 2410, Mbeya, Tanzania, Email: nimr-mmrc@nimr-mmrc.or

NIMR Muhimbili, P.O. Box 3436, Dar es Salaam, Tanzania, Email: muhimbili@nimr.or.tz

NIMR Mwanza, P.O. Box 1462, Mwanza, Tanzania, Email: mwanza@nimr.or.t

NIMR Ngongongare, P.O. Box 514, USA River, Arusha, Tanzania, Email: ngongongare@nimr.or.tz

NIMR Tabora, P.O. Box 482, Tabora, Tanzania, Email: tabora@nimr.or.tz

NIMR Tanga, P.O. Box 5004, Tanga, Tanzania, Email: tanga@nimr.or.tz

Response: Thank you for noting this. We have improved the text to address this comment.

lines - 418-437 - duplicated?

Response: We have removed the duplicate information from this revised manuscript.

line 438 - commented, not commended?

Response: Thank you for the observation. We have improved the overall grammar of the manuscript.

2. The use of the Sekhon et al. framework on acceptability as a conceptual framework is appropriate. However, it's introduction to the reader (lines 171-188) lacks clarity. Further, the operationalization of the key concepts to your data requires additional explanation. It's good to use this to organize the findings in principle, but in practice the findings seem very labored in presentation. Perhaps the acceptability concepts could be reframed as questions of the data, used as subheadings to the findings (e.g., Did the intervention 'make sense' (coherence) to participants?). These can then be answered more declaratively with "Yes", or "Yes, most of it, but not ... " and so on.

Response: Thank you for your comment. We have revised the description of the theoretical framework of acceptability and operationalized its key concepts for improved clarity. Furthermore, we have reorganized the presentation of the findings. We now describe themes related to the TFA constructs in two broad categories: factors influencing acceptance of the

family approach and barriers to acceptance, which can be targeted for improved acceptance.

3. The discussion is somewhat speculative. How much more do we really learn about TPT support for PLHIV from these data? The novelty should be in the combining of support elements (treatment support, sms etc.) and the overall acceptability of this combined approach. Instead, much of the discussion is (a) about each element in isolation, and (b) quite equivocal about what was learned. The discussion and then conclusion can be made more punchy once these comments are addressed.

Response: Thank you for the comment. We have revised the discussion section to provide meaningful information on the overall acceptability of the family approach as a combined approach. This has also resulted in a revision of the conclusion.

1) Can you describe the role of the community health workers (CHW's) in this program? It is unclear from the description what role the CHW's play in prescribing, educating, sending SMS messages and supporting other aspects of the program.

Response: Thank you for the comment. We have described the role of CHWs in lines 167 to 169 on the revised clean version of the manuscript.

2) Who is sending the SMS reminders? This is not clear in the description of the program.

Response: Thank you for this note. In lines 154 to 156, we describe the sender of the SMS to PLHIV and their TS.

3) The manuscript would be substantially strengthened by a summary set of recommendations on how the program should be re-designed based on this study. For example, should there be fewer than 3 training sessions? Should any other refinements be made?

Response: Thank you. We concur with this observation. We have highlighted a set of recommendations for programmatic adoption.

Minor corrections:

1) Page 5 line 119 – 'hypertoxicity' I think is meant to be 'hepatotoxicity'

Response: Thank you. We have corrected the word.

2) Page 12, Table 1: There is no data in the row 'CTC working experience (median, IQR)

Response: Thank you for bringing this to our attention. It was an overlook on our part. We have removed the row as it pertains to a different set of participants addressing a separate objective.

Yours sincerely,

Doreen Pamba
