# Supplementary material for: Optimizing delivery strategies for 3HP TB preventive treatment in Tanzania: A qualitative study on acceptability of family approach in HIV care and treatment centers
Source: PLOS Glob Public Health. 2024 Dec 17;4(12):e0003142. doi: 10.1371/journal.pgph.0003142 (PMC11651582; doi:10.1371/journal.pgph.0003142)
Supplement: Attachment — Submitted filename: 2nd Response to Reviewers.pdf [file pgph.0003142.s006.pdf]

THE UNITED REPUBLIC OF TANZANIA

MINISTRY OF HEALTH

NATIONAL INSTITUTE FOR MEDICAL RESEARCH

Date: 1st November 2024

Dear Editor(s)

PLOS Global Public Health

The authors of this manuscript would like to thank you and the reviewers for dedicating time to review our work and for providing constructive feedback. Your input has significantly improved our scientific writing and the manuscript's content. Below is a thorough point-by-point response to each editorial point raised by the first reviewer. All revisions made to the manuscript are indicated using tracked changes.

Comment #1: Thank you to the authors for addressing my previous comments. I still believe that the findings could be presented more succinctly, with shorter quotes, or perhaps having the quotes in a table.

> *Response: Thank you for pointing this out. We have shortened the quotes to ensure that the study findings are presented briefly and clearly.*

Comment #2: Also, there are still several typos throughout, e.g.: line 138 - sub-Saharan Africa, not Sub-Sahara Africa, or lines 172-174, the citation (19) should appear here, not after the next sentence, or line 199 surely 3HP TPT or just 3HP rather than 3HPTPT? But, overall, the manuscript is coherent and reports important data.

> *Response: This is very valid. Thank you for noting this. We have addressed the typos and improved the manuscript's overall grammar. Additionally, we could not locate a typo in line 199 as in the 2nd version of*

Headquarters: 3 Barack Obama Drive, P.O. Box 9653, 11101 Dar es Salaam, Tanzania,
Phone: +255222121400, Email: info@nimr.or.tz, Website: www.nimr.or.tz

NIMR Amani
P.O. Box 81
Muheza, Tanga
Tanzania
Email: amani@nimr.or.tz

NIMR Dodoma
Benjamin Mkapa Hospital
P.O. Box 805
41119 Dodoma, Tanzania
Email: dodoma@nimr.or.tz

NIMR Mbeya
Hospital Hill Rd
P.O. Box 2410, Mbeya,
Tanzania
Email:
nimr-mmrc@nimr-mmrc.or

NIMR Muhimbili
P.O. Box 3436
Dar es Salaam, Tanzania
Email:
muhimbili@nimr.or.tz

NIMR Mwanza
P.O. Box 1462
Mwanza
Tanzania
Email: mwanza@nimr.or.t

NIMR Ngongongare
P.O. Box 514
USA River, Arusha
Tanzania
Email: ngongongare@nimr.or.tz

NIMR Tabora
P.O. Box 482
Tabora
Tanzania
Email: tabora@nimr.or.tz

NIMR Tanga
P.O. Box 5004
Tanga
Tanzania
Email: tanga@nimr.or.tz

THE UNITED REPUBLIC OF TANZANIA

MINISTRY OF HEALTH

NATIONAL INSTITUTE FOR MEDICAL RESEARCH

the manuscript submitted in response to reviewer comments, only 3HP was stated and not 3HP TPT. The typo was, however, present in the first version of the manuscript that was uploaded during the initial submission

Yours sincerely,

Doreen Pamba

All official correspondences should be addressed to the Director General

Headquarters: 3 Barack Obama Drive, P.O. Box 9653, 11101 Dar es Salaam, Tanzania,
Phone: +255222121400, Email: info@nimr.or.tz, Website: www.nimr.or.tz

NIMR Amani
P.O. Box 81
Muheza, Tanga
Tanzania
Email: amani@nimr.or.tz

NIMR Dodoma
Benjamin Mkapa Hospital
P.O. Box 805
41119 Dodoma, Tanzania
Email: dodoma@nimr.or.tz

NIMR Mbeya
Hospital Hill Rd
P.O. Box 2410, Mbeya,
Tanzania
Email:
nimr-mmrc@nimr-mmrc.or

NIMR Muhimbili
P.O. Box 3436
Dar es Salaam, Tanzania
Email:
muhimbili@nimr.or.tz

NIMR Mwanza
P.O. Box 1462
Mwanza
Tanzania
Email: mwanza@nimr.or.t

NIMR Ngongongare
P.O. Box 514
USA River, Arusha
Tanzania
Email: ngongongare@nimr.or.tz

NIMR Tabora
P.O. Box 482
Tabora
Tanzania
Email: tabora@nimr.or.tz

NIMR Tanga
P.O. Box 5004
Tanga
Tanzania
Email: tanga@nimr.or.tz
