# Supplementary material for: Optimizing delivery strategies for 3HP TB preventive treatment in Tanzania: A qualitative study on acceptability of family approach in HIV care and treatment centers
Source: PLOS Glob Public Health. 2024 Dec 17;4(12):e0003142. doi: 10.1371/journal.pgph.0003142 (PMC11651582; doi:10.1371/journal.pgph.0003142)
Supplement: Attachment — Submitted filename: 2024_PLOSGlobalHealth_TPT in TZ.docx [file pgph.0003142.s004.docx]

**Review for PLOS Global Public Health “Optimizing delivery strategies for 3HP TB preventive treatment in Tanzania: A qualitative study on acceptability of family approach in HIV care and treatment centers”**

Thank you for the invitation to review this very interesting manuscript on a qualitative assessment of the acceptability of a family approach to the 3HP TPT in Tanzania. The manuscript is well-written and interesting.

Major concerns:

1) Can you describe the role of the community health workers (CHW’s) in this program? It is unclear from the description what role the CHW’s paly in prescribing, educating, sending SMS messages and supporting other aspects of the program.
2) Who is sending the SMS reminders? This is not clear in the description of the program.
3) The manuscript would be substantially strengthened by a summary set of recommendations on how the program should be re-designed based on this study. For example, should there be fewer than 3 training sessions? Should any other refinements be made?

Minor corrections:

1) Page 5 line 119 – ‘hypertoxicity’ I think is meant to be ‘hepatotoxicity’
2) Page 12, Table 1: There is no data in the row ‘CTC working experience (median, IQR)
